# Web Alert: Commercial probiotic microorganisms

**DOI:** 10.1111/1751-7915.14120

**Published:** 2022-07-27

**Authors:** Lawrence P. Wackett

**Affiliations:** ^1^ Department of Biochemistry, Molecular Biology & Biophysics, BioTechnology Institute University of Minnesota, St. Paul MN USA

Diversity of commercial probiotic strains.

https://www.ncbi.nlm.nih.gov/pmc/articles/PMC6430388/

In response to incomplete labels, this study cultured common strains from 21 different commercial probiotic preparations and sequenced the genomes.

Viability of probiotic strains.

http://www.ijpmbs.com/uploadfile/2020/0622/20200622030828608.pdf

This study investigated the ability of microbes in probiotic products to survive strong acid conditions. This was done to learn about potential survival in the acid portions of the digestive tract.

Probiotics: NIH.

https://www.nccih.nih.gov/health/probiotics‐what‐you‐need‐to‐know

This is a basic, but useful, site on all aspects of probiotics, bacteria, products, and usage statistics. It is put out by the U.S. National Institutes of Health.

Whole genome analysis of probiotic strains.

https://bmcgenomics.biomedcentral.com/articles/10.1186/s12864‐021‐07539‐9

This study examined the genes in the genomes of commercially‐relevant probiotic bacterial strains. The focus was on their identification of genes putatively involved in toxin production, other virulence factors, and antibiotic resistance.

Comparative genomics of probiotic strains.

https://www.frontiersin.org/articles/10.3389/fmicb.2019.00712/full

This study looked at genomes to gain better insights into species diversity, risks and impacts of traditional probiotic microbial species on gut microbiomes.

Probiotics and metabolic landscapes.

https://www.biorxiv.org/content/10.1101/2021.06.20.449192v1.full

This study looks at the genomes and metabolism of probiotic lactic acid bacteria. Lactic acid bacteria are defined as members of the following genera: *Lactobacillus, Lactococcus, Leuconostoc, Oenococcus, Pedicoccus, and Streptococcus*.

Gut persistence of a probiotic strain.

https://journals.asm.org/doi/10.1128/JB.01637‐07

A major goal of probiotic formulations is to provide some lasting benefits to gut microbiomes. This study dealt with the identification of genes associated with long‐term persistence in the gut.

How probiotics affect the microbiota.

https://www.frontiersin.org/articles/10.3389/fcimb.2019.00454/full

This is a review article on the basics of probiotics with an emphasis on their effects on the gut microbiota.

Regulating microbiome manipulation.

https://www.nature.com/articles/s41591‐019‐0451‐1

This comprehensive review discusses both over the counter probiotics and those prescribed for specific diseases. It takes the view that potential risks are largely being overlooked.

Global probiotics market.

https://www.prnewswire.com/news‐releases/at‐8‐4‐cagr‐probiotics‐market‐size‐is‐expected‐to‐reach‐usd‐108‐16‐billion‐to‐2028‐by‐globally‐says‐brandessence‐market‐research‐301497707.html

This site reports the global value of the probiotic business market, expected growth, and major companies involved. It also provides links to further information.

